# Analogue information processing in NF-κB gene regulatory system

**DOI:** 10.1098/rsos.241487

**Published:** 2025-06-04

**Authors:** Pankaj Gautam, Dinesh Kashyap, Debabrata Biswas, Sudipta Kumar Sinha

**Affiliations:** ^1^ Department of Chemistry, Indian Institute of Technology Ropar, Rupnagar, Punjab, India; ^2^ Department of Physics, Bankura University, Bankura, West Bengal, India

**Keywords:** biochemical networks, bifurcation theory, Fokker–Planck equation, stochastic gene expression, coloured noise, time delay

## Abstract

Nuclear factor-κB (NF-κB) participates in various cellular processes to encompass cell fate through differential gene expression, but the underlying molecular mechanism behind this phenomenon is still elusive. Two factors in this process can control the gene expression for determining the cell’s fate: (i) synthesized proteins may have a considerable lifetime and (ii) gene activation may be slow or delayed. To address the first factor, we argue that the NF-κB system experiences cellular variability, often considered the origin of environmental noise for protein production, which influences cellular decisions at the molecular level as they have a considerable lifetime. We employ unified coloured noise approximation to obtain analytical expressions for the protein mean number obtained from our theoretical model and stochastic simulation. We find that these fluctuations influence mean protein numbers and induce bimodality. However, for the second factor, we rely on experimental findings, where the time delay in gene activation plays an essential role in protein production. Our bifurcation analysis demonstrates that the system exhibits saddle-node bifurcations for the instantaneous case, but it experiences the Hopf bifurcation and oscillates between two states in the presence of the time delay. In a nutshell, as NF-κB dynamics influence downstream expression, this study may provide insight into how to adjust parameters to control gene expressions.

## 1. Introduction

The NF-κB, a family of dimeric transcription factors (TFs), has a crucial role in regulating several cellular processes that include the immunity, inflammation and development of tumours [1–4]. The translocation of NF-κB occurs into the nucleus, which regulates the transcription of the downstream anti-apoptotic, pro-apoptotic and proinflammatory target genes [5,6]. It acts as a typical activator that regulates antigen-dependent B-cell differentiation either via direct binding to a regulatory region of a DNA for enhancer activity, chromatin opening or regulating the RNA expression in a single cell. This TF forms the complex with another protein, namely IκBα, on phosphorylation, and it plays a crucial role in processing the proinflammatory inputs by responding to many pathogenic and self-signals [7]. Tumour necrosis factor alpha (TNF-α) or lipopolysaccharide (LPS) acts as an inflammatory signal that induces phosphorylation, ubiquitination and degradation of IκBα proteins [8]. Upon binding to the promoter or enhancer target genes, NF-κB can regulate many chemokines, inflammatory factors, stress response genes and hundreds of other target genes [9–11]. Previous studies [12–14] reported the experimental evidence that, first, in most cases of human tumours, the deregulation of NF-κB occurs, and second, inhibition of NF-κB pathway blocks the cell cycle and apoptosis is induced.

The gene regulatory circuit of NF-κB exhibits molecular memory [15], and its role in the expression remains unclear. Numerous experiments demonstrate that NF-κB oscillates between an inactive form outside of the cell’s nucleus and an active form inside, based on the signals a cell receives from outside, thereby helping NF-κB to display significant functions in inflammation and cancer [16,17]. Furthermore, some genes turned on by the active form of NF-κB generate biomolecules that inactivate NF-κB, which sets up the oscillations. Based on the variety in the regulatory mechanisms of NF-κB-mediated gene activation and the establishment of oscillatory behaviour could play some physiological role in the gene regulation, which remains to be understood [17]. Tay *et al*. [18] showed that cells encode an intricate set of analogue parameters to fine-tune the gene expression outcome; these parameters include NF-κB peak intensity, response time and number of oscillations. Hoffmann *et al*. [19] observed oscillations in the nuclear-cytoplasmic translocation of the NF-κB and demonstrated that negative feedback regulation leads to oscillatory NF-κB activation profile. Moreover, IκBβ and the noise strength (ε) respond slowly to IκB kinase (IKK) activation and act to dampen the long-term oscillations of the NF-κB response. Krishna *et al*. [20] examined the small core network consisting of cytoplasmic and nuclear NF-κB, its inhibitor, IκB and IKK drives the oscillations, which is built to produce periodic spikes in nuclear NF-κB concentration. However, exclusive inclusion of the time delay in gene expression alone can cause the oscillatory behaviour, and we show how parameters and noise in this study influence the bimodality.

The oscillation may also arise due to the delayed gene activation [21,22]. Indeed, it has been shown experimentally that gene activation is a time-delayed phenomenon [23], which is attributed to the intricate NF-κB binding to the promoter DNA that requires special attention for modelling [24–26]. Moreover, the biological function of NF-κB is complex, generating a wide range of cellular variability in response to stimuli such as TNF-α, LPS, etc. [15,27,28]. Such stimulation of NF-κB depends on the intrinsic stochasticity over a broad spectrum of noises governed by various biomolecular events [16,18,29,30]. Cellular systems also experience external noise governed by changes in environmental conditions such as temperature fluctuations, cell volume, etc. [30–32]. However, a few correlated events, as governed by coloured noise, impact the genotype-to-phenotype changes and determine cellular variability [33–35]. The NF-κB promotes the expression of IκBα protein, and various cytokines stimulate the IκB kinases that phosphorylate the IκB proteins, leading to their degradation. A recent report shows that the downregulation of IκB kinases (IKKα and IKKβ) is associated with NF-κB transactivation, evidenced by a time-dependent degradation of IκBα by an upstream path [36]. The existence of time-dependent degradation of the protein may be attributed to the fact that the IκBα experiences correlated noise, and therefore, the protein is non-transient and possesses a lifetime [36,37]. Thus, one can employ coloured noise formalism to investigate the effects of a time-varying degradation process involving a multistep IκBα protein degradation mechanism.

Single-molecule imaging is a powerful tool that captures signals from a noisy environment in the live cell, but their theoretical modelling is still challenging [34,38]. The mass action kinetics-based deterministic models may be a starting point for understanding the dynamics of a biochemical network, which offers to understand the existence of alternative steady states [39,40]. These dynamical models are typically nonlinear. The analytical solution corresponding to the gene regulatory networks (GRNs) having a few molecular components is reported in the literature by solving chemical master equations using the generating function method, self-consistent proteomic field, binomial moment, etc. Despite its significance, the analytical methods are unable to handle intricate regulatory systems having many nonlinear functionalities. The bifurcation theory can help to study the stability of such dynamic nonlinear systems [41]. Therefore, one can also develop stochastic models of this network by incorporating external or internal noises. The noise can drive transitions from one steady state to another alternative state, a feature absent in deterministic systems. These stochastic models have broad applicability, from ecological to gene regulatory systems operating at diverse timescales [42,43]. Since the system experiences broad-spectrum noises, one must carefully incorporate them for appropriate modelling. The noise sources are typically from various stochastic events linked with gene expression, cellular responses to external stimuli or immune responses. Therefore, theoretical modelling approaches may be an avenue to explore, but obtaining a closed-form analytical solution for such a complex system remains a challenging problem [34,38]. Stochastic simulation algorithms (SSA) provide a brute-force method to model them based on the elementary realized reactions for gene regulation [44]. These modelling schemes are system-specific, and often they experience a broad spectrum of noises ranging from delta function correlated white noise to the correlated coloured noise [35,45].

As mentioned before, another aspect of the NF-κB system is the delayed gene activation, as exhibited in this system. The inclusion of time delay makes the system infinite-dimensional and causes the system to experience oscillation originating from Hopf bifurcation depending upon the delay [22,46]. Thus, the oscillation of NF-κB (and IκBα) provides a clue that the cell may suffer from extracellular stimulation of diseases like cancer [12,16]. Oscillations of NF-κB may be attributed to the fact that temporal code conveys the information about the stimulus to gene promoters [16]. A large number of past works on NF-κB oscillations are mainly focused on the negative feedback loops and the instantaneous transcriptional and translational phenomena, but the time required for these gene activation processes has not been studied precisely. The typical delay in the transcription process is of the order of 10–20 min and for translation approximately 1–3 min [21,24,47]. Further, experimental studies suggest that the time delay may be varied. Reference [48] reported that the time delay reduces by 19 min if all three introns within the Hes7 gene are removed and oscillatory expression is suppressed completely. Thus, the IκBα protein synthesis time delay may be controlled through the intron splicing within the genes of IκBα. The existing research suggests that the existence of time delay may lead to oscillatory and complex dynamical behaviours in GRNs [49–52]. Thus, the delay in protein synthesis is standard, and it is ubiquitous to consider a delay in the model of the gene regulation network.

These oscillating concentrations of nuclear NF-κB (and IκBα) play crucial roles in several downstream gene expressions, resulting in different fates for cells, leading to phenomena like proliferation and apoptosis [16,53]. Hence, NF-κB (and IκBα), due to its competency to promote the survival of the cancer cells and apoptosis, plays a dual role in carcinogenic and anti-tumour capabilities. Firstly, the damaged cells will be programmed to death if the activation of the downstream pro-apoptotic factors (e.g. TNF, Bax, c-myc, etc.) occurs. Hence, this will avoid the inheritance of damage and wrong information to the daughter cells of the next generation. Thus, the oscillation of NF-κB (and IκBα) plays a vital role in the impression of cancer [9,54]. Secondly, the damaged cells will survive if the activation of the downstream anti-apoptotic genes (e.g. TRAF, c-iAP, etc.) occurs. Thus, the cells of the next generation are unhealthy also, whose development may lead to inflammation as well as cancer [12–14,55,56]. So the oscillation of NF-κB (and IκBα) plays a vital role in inflammation and cancer by determining the downstream gene expressions and cell fate choices [16,17,19,29,57–59]. Thus, the study of NF-κB (and IκBα) oscillation and its control poses a critical research aspect, which is little known.

In this work, we propose a new model for the regulation of NF-κB-mediated transcription of inflammatory genes (e.g. TNF, IRF1, IKBA genes, etc.) to describe the time-delayed dynamics and the dynamics in the presence of coloured noise. The proposed model explores the NF-κB-dependent switching between genotype to phenotype states as described by multiple steady states. Although stochastic biomolecular interactions are responsible for fine-tuning the expression output by modulating various regulatory pathways, this work shows how extrinsic noise can control the bistability feature for the NF-κB-dependent GRN. The model offers flexibility or an alternative regulatory pathway for phenotypic switching, which does not require biomolecular cooperative interactions. We analyse the bifurcation diagram of the system for both instantaneous time and time-delayed cases. To incorporate short memory, we consider the time-varying rate parameters as defined by an Ornstein–Uhlenbeck (OU) process that affects the degradation of the IκBα protein. We analyse them theoretically as well as by simulation. To analyse this model, we adopt the unified coloured noise approximation (UCNA) to deal with the short molecular memory observed during protein degradation [45,60]. In the case of time delay, the delay-induced oscillations come into play for the overall dynamics of the system. The importance of oscillation in NF-κB and IκBα is discussed in the subsequent sections. Overall, we specifically aim (i) to develop a general protocol to obtain the steady state distribution of IκBα protein, where the NF-κB regulates through a negative feedback loop, (ii) to study the time-delay dynamics of NF-κB (IκBα) controlled gene responses that show oscillatory behaviours, (iii) to observe the effect of time-varying rate parameter by considering correlated noise and to calculate power spectral densities for such systems to observe the relation between the frequency spectra and the coloured noise for this NF-κB regulatory circuit, and (iv) to explore the quantitative distinction between typical enhancer (TE) and super-enhancer (SE)-mediated gene expressions, in this work.

## 2. Methods

### 2.1. Theoretical approaches

**Figure 1.**
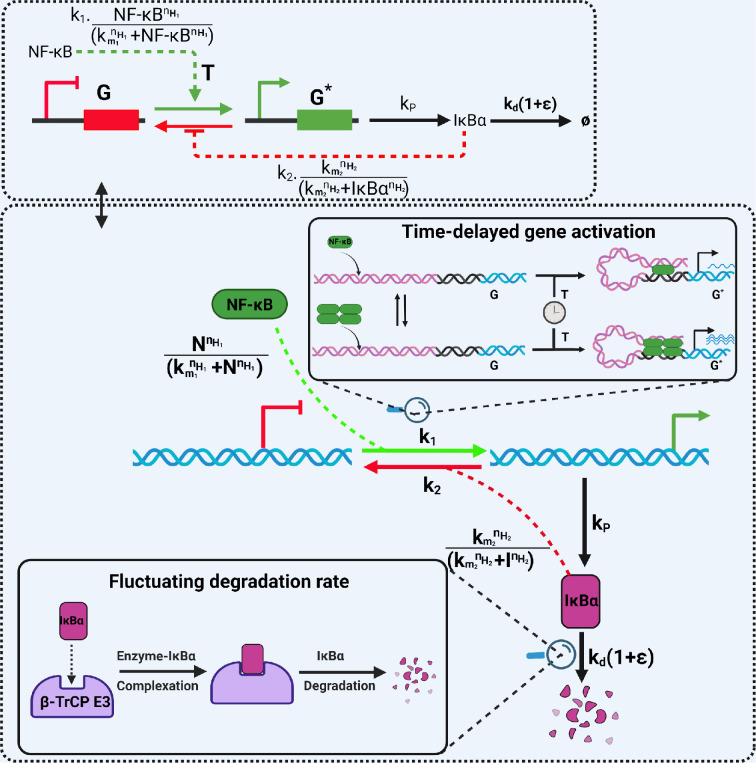
The schematic view of the negative feedback loop between the inactive and active genes and their regulation is controlled by NF-κB and IκBα proteins. The circuit diagram for the NF-κB-mediated gene regulation for producing IκBα has been in the top box. The respective model equations (2.2a,b), (2.4a) and (2.4b) are presented in §2. Various molecular events for this regulation are shown in the bottom dashed box. In the inset of the bottom dashed box, the time-delayed gene activation that happens due to the long-distance enhancer–promoter interaction through DNA looping is shown. In the left inset of the bottom dashed box, we show the fluctuation in protein degradation, which one can realize because the particular step proceeds in a multistep manner. As shown in the figure, the degradation of IκBα protein follows a complex-forming path through the binding with E3 ligase β-transducin repeat-containing proteins (β-TrCP E3).


We employ a simple model for the gene activation (deactivation) mechanism of the NF-κB system as a guideline. However, it maintains a high degree of biological relevance for gene regulation in similar systems of interest. For this we follow the works reported by Michida *et al.* [61] and Zambrano *et al.* [17]. The schematic of the whole reaction is summarized in figure 1. The dynamics of this system were analysed deterministically as well as stochastically. The details of the analyses are presented in the electronic supplementary material. In the modelling process, we express the biochemical reaction scheme for the system as follows.

(2.1a)G→k1fA(NF−κB)G∗,G∗→k2fR(Iκ Bα)G,(2.1b)G∗→kPG∗+IκBα,IκBα→kdϕ

where G and $G^{*}$ are the genes in inactive and active states encoding IκBα protein; $\{k_{1}$, $k_{2}$, $k_{P}$, $k_{d}\}\in R^{+}$ are the respective rate constants for the reactions. The TF, NF-κB, starts transcription by binding the promoter for producing IκBα protein. Here, we are considering the role of IκBα as a transcriptional repressor, wherein IκBα binds to specific genomic regions with its recruitment to the promoter responsible for regulating gene transcriptional output [62–64]. We assume that NF-κB can be either free or bound to IκBα to form the complex (NF-κB : IκBα). The free NF-κB can be assumed to be nuclear and will control the activation of the inactive gene (G) to the activated gene (G${,}^{*}$). In contrast, the complex (NF-κB : IκBα) is cytoplasmic and is thus inactive towards the modulation of the gene activation process [17]. We refer to NF-κB and IκBα as N and I, respectively. The deterministic rate equations for this system model are given by


(2.2a)dG∗dt=k1fA(N)G−k2fR(I)G∗,(2.2b)dIdt=kPG∗−kdI.

Here, $G+G^{*}\;=1$, $f_{A}(N)=N^{n_{H_{1}}}/(K_{m_{1}}^{n_{H_{1}}}+N^{n_{H_{1}}})$ and $f_{R}(I)=K_{m_{2}}^{n_{H_{2}}}/(K_{m_{2}}^{n_{H_{2}}}+I^{n_{H_{2}}})$, where $\{K_{m_{1}},K_{m_{2}}\}\in R^{+}$ stand for the binding affinity of NF-κB and IκBα, respectively, and $\{n_{H_{1}},n_{H_{2}}\}\in R^{+}$ are the Hill’s coefficients given for the binding of NF-κB and IκBα to their respective genomic regions. One can realize the degree of binding of these proteins to the respective genomic regions embedded in $n_{H_{1}}$ and $n_{H_{2}}$. We first assume that the gene activation and deactivation are fast. Therefore, one may apply quasi-steady state approximation (QSSA). Immediately, the corresponding deterministic rate equation for mean IκBα protein number (I) under QSSA curtails to the form:


(2.3) $$\frac{dI}{dt}=\frac{k_{1}k_{P}f_{A}(N)}{k_{1}f_{A}(N)+k_{2}f_{R}(I)}-k_{d}I.$$

Equation (2.3) comes from the QSSA, where we assume that the free NF-κB can be nuclear and will control the activation of the inactive gene (G) to activate gene (G${,}^{*}$) rapidly. Since the process is relatively fast, we can apply QSSA approximation to reduce the dimension of the dynamical equation.

However, many experiments demonstrate that producing the IκBα is an intricate biochemical process that requires a certain amount of time [21,24]. The formation of the complex (NF-κB : IκBα) is cytoplasmic and is thus inactive towards the modulation of the gene activation process. This statement is supported by mathematical models [65] and by experiments [17]. Furthermore, it is suggested by the literature, both in mathematical models [18] and experimentally [66], that the presence of nuclear IκBα can trigger detachment of NF-κB from the promoter and thus the inactivation of the gene. While equation (2.3) came into our study under the assumptions of fast activation and deactivation mechanisms that happen through NF-κB and IκBα binding.This assumption is not very practical for the NF-κB signalling system since there exist two reaction channels that can enhance the population of the long-lived NF-κB : IκBα complex inside the nucleus [67]. These two channels are that (i) the NF-κB can bind exclusively with IκBα and form NF-κB: IκBα stable complex in the nucleus [67], and (ii) the increase of the concentration of IκBα inside the nucleus promotes deactivation of G∗ upon binding with the NF-κB, which further enhances the population of the NF-κB : IκBα complex inside the nucleus [67]. Thus, the previous model equation (2.3) has some shortfalls, which we overcome by assuming that the stable NF-κB : IκBα complex dissociates after a delay period (T), which again binds with the κB response element for the activation. To incorporate such an effect, we introduce the delay for gene activation into the dynamical equation. Typically, the average transcriptional delay time is approximately 10−20 min, which implies that the gene activation is a delayed process for this system [21,47,68]. Therefore, we consider a model that incorporates the time delay for the NF-κB-mediated gene activation [22,62,63,68]. For finite time-delay ranges, we calculated the IκBα protein production levels by assuming that the delayed gene activation occurs through the formation of NF-κB–DNA complex, which may be considered as delayed gene activation [21,22]. Assuming this, we include the time delay in the system equation (2.2) and write it in the following way:


(2.4a)dG∗dt=k1fA(N)G(t−T)−k2fR(I)G∗,(2.4b)dIdt=kPG∗−kdI,

where $G(t-T)=1-G^{*}(t-T)$ is the delayed state. $T\in R^{+}$ is the finite positive time delay involved in the gene activation process. Other variables and parameters have been described in the §2.1.

The analysis based on the above kinetic equations is limited in its ability to characterize probabilistic events, as they do not capture multimodality in gene expression that arises from slow promoter binding [45,69]. Therefore, one can consider the birth and death processes of the networks’ elementary reactions. Generally, it is described by the time evolution of a grand probability function governed by a chemical master equation. Further, the master equation can be reduced to the chemical Fokker–Planck equation (FPE) by the Stratonovich sense [70], which allows one to calculate the probability, P(I,t):


(2.5) $$\frac{\partial }{\partial t}P(I,t)=-\frac{\partial }{\partial I}[a_{1}(I)+a_{2}(I{)}^{2}P(I,t)]+\frac{1}{2}\frac{\partial^{2}}{\partial I^{2}}[a_{2}(I)P(I,t)],$$

where $a_{1}(I)$ and $a_{2}(I)$ are the first two jump moments, written as $a_{1}(I)=S^{+}(I)+S^{-}(I)$ and $a_{2}(I)=S^{+}(I)-S^{-}(I)$, respectively. The steady-state solution of the FPE has the form [31]


(2.6) $$P(I)=\frac{Q}{\sqrt{a_{1}(I)}}exp\left(2\int^{I}\frac{a_{2}(I)}{a_{1}(I)}\right)dI,$$

where Q is a normalization constant, and $S^{+}(I)=\frac{k_{1}k_{P}f_{A}(N)}{k_{1}f_{A}(N)+k_{2}f_{R}(I)}$ and $S^{-}(I)=k_{d}I$.

Since the degradation of IκBα protein is slow compared to the other TF-DNA-binding/unbinding events [23], it is natural to introduce a correlated process for it [20,37]. In our analysis, we include the effect of such slow decay on protein degradation by introducing short-range correlated noise to the degradation parameter $k_{d}$. Technically, we consider fluctuating ${\tilde{k}}_{d}$ driven by the OU process [45,71]. We define ${\tilde{k}}_{d}$ as $k_{d}+k_{d}\epsilon$, where $k_{d}$ is the mean value and ϵ is the Gaussian correlated noise (colour) defined by the OU process, $\frac{d\epsilon }{dt}=-\frac{\epsilon }{\tau }+\sqrt{\frac{2}{\tau }}\xi (t)$, where ξ(t) is a zero-mean Gaussian white noise with $\langle \xi (t)\xi (t^{{,}^{\prime }})\rangle =2D\delta (t-t^{{,}^{\prime }})$. With this definition, the Gaussian colour noise, ϵ(t) has zero mean and correlator $\langle \epsilon (t)\epsilon (t^{\prime })\rangle =\frac{D}{\tau }e^{-\frac{|t-t^{\prime }|}{\tau }}$. With this correlated process, the chemical FPE equation can be written in the Stratonovich form [45,70] as


(2.7) $$\begin{array}{lrr} & \frac{\partial }{\partial t}P(I,t)=-\frac{\partial }{\partial I}[(\tilde{h}(I)+\tilde{g}(I{)}^{2})P(I,t)]+\frac{\partial^{2}}{\partial I^{2}}[\tilde{g}(I{)}^{2}P(I,t)] & \end{array}$$

Here, $\tilde{h}(I)=h(I)/Z$ and $\tilde{g}(I)=g(I)/Z$. Immediately, the corresponding steady-state solution is written as


(2.8) $$\begin{array}{lcr} & P(I)=\frac{Q}{\tilde{g}(I{)}^{2}}exp\left(\int^{I}\frac{\tilde{h}(I)+\tilde{g}(I){\tilde{g}}^{{,}^{\prime }}(I)}{\tilde{g}(I{)}^{2}}\right)dI, & \end{array}$$

where the Q is the normalization constant, $h(I)=a_{2}(I)$, $g_{1}(I)=-S^{-}(I)$, $g_{2}(I)=\sqrt{a_{1}(I)}$ and ξ(t) is a zero-mean Gaussian white noise with $\langle \xi (t)\xi (t^{{,}^{\prime }})\rangle =2D\delta (t-t^{{,}^{\prime }})$. The detailed theoretical analyses corresponding to the inclusion of coloured noise in the degradation rate parameter are presented in the electronic supplementary material, section II.C.

#### 2.1.1. Stochastic simulation

Despite their importance, analytical methods cannot tackle complex regulatory systems involving many nonlinear functioning promoters. Therefore, one can perform stochastic simulation by considering each elementary reaction of each network. The stochastic dynamics directly provide a signature on the robustness and stability of each network motif. We use the Gillespie algorithm to perform our stochastic simulations [44]. It provides a realistic view of where the fluctuations in the abundance of the molecules in living cells affect their growth. Geometrically, the number of molecules changes to the random walk on a multidimensional state space [31]. Stochastic simulation has an advantage over its deterministic version because the former method takes care of the system’s intrinsic fluctuations, allowing the system’s state to switch from one to another. The trajectories obtained from stochastic simulations sample the Markov process as realized by a set of elementary reactions whose joint distribution is described by the master equation. The simulation details and parameters used for this simulation are presented in the electronic supplementary material, section II.E.

## 3. Results and discussion

We analyse both deterministic and stochastic models systematically. We first perform the bifurcation analysis of the deterministic model as a function of the degradation parameter ($k_{d}$), NF-κB level (N), and activation parameter ($k_{1}$) for the protein production. The analysis is done using the continuation package XPPAUT [72], and we consider $k_{1}=1$, $k_{2}=3.4$, $k_{p}=0.065$, $k_{d}=0.002$, $K_{m_{1}}=K_{m_{2}}=12$, $n_{H_{1}}=1$, $n_{H_{2}}=4$ and N=100 unless otherwise used as the control parameters in the bifurcation. We further perform the bifurcation analysis of the delayed system, where the gene activation occurs through slow promoter binding. We marked the territories for the existence of oscillatory and stable steady-state regions for the variation of the control parameters $k_{d}$, and the time delay T. We compute the steady-state probability distribution (SSPD) from our stochastic models. The calculations are also done in the presence of short-range correlated noise in the degradation parameter $k_{d}$. Finally, we present the power spectral densities obtained from the stochastic trajectories.

### 3.1. Existence of multiple steady states

#### 3.1.1. Non-delayed dynamics: occurrence of bistability

We first conduct the bifurcation analysis of our model A bifurcation diagram quantifies how the behaviour of a dynamical system changes in the long term as a function of a parameter. In other words, it describes a change in the stability or existence of fixed points as the system parameters change. In a biological system, such perturbation in a parameter is inevitable since we observe sudden switching among stable steady states as phenotype diversity.

**Figure 2.**
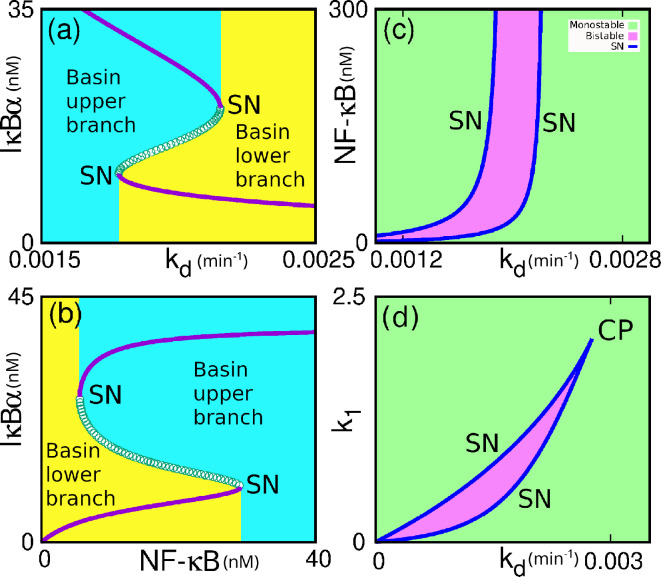
Single-parameter bifurcation diagrams for the control parameters $k_{d}$, and NF-κB showing the occurrence of saddle-node bifurcations and bistability in the system in the panels (a) and (b). Yellow and cyan colours show the basin of attraction for the lower and upper branches of fixed points, respectively. (c) Two-parameter bifurcation diagram in $k_{d}-$NF-κB space, showing the bistable zone originating from saddle-node bifurcations, separating two monostable zones. (d) Two-parameter bifurcation diagram in $k_{d}$-$k_{1}$ space, showing the bistable zone originating from saddle-node bifurcations, separating two monostable zones. This figure shows the concentration of NF-κB and the rate of degradation of IκBα protein ($k_{d}$) induced saddle-node bifurcation for the number of IκBα species. We also have shown the presence of a *cusp point* for these parameters, which shows a boundary between monostable and bistable fixed points.

Figure 2a,b shows the population of IκBα with the bifurcation parameters, $k_{d}$ and NF-κB, respectively. It suggests that the system exhibits bistability, as evident from the saddle-node (SN) or fold bifurcation. The existence of SN bifurcation demonstrates the hysteretic behaviour of the system, as evident from figure 2. From panel (a), we see an increase of $k_{d}$ from low to high values causing the system to switch from the upper branch to the lower one. But (b) suggests that increasing NF-κB causes the system to swift from the lower to the upper branch. It is clear from the figure that the system exhibits bistability only over a range of parameter values, and it experiences monostability in other regions. Literature suggests that bistability (or, in general, multistability) is an essential recurring theme for understanding various cell signalling or cellular functioning, which includes decision-making biophysical processes such as cell cycle progression, cell differentiation and apoptosis [73–75]. These observations from panels (a) and (b) shed light on the presence of the two stable steady states and the existence of the bimodal distribution in the population of IκBα influenced by the rate constant $k_{d}$ and NF-κB, respectively, in our system.

**Figure 3.**
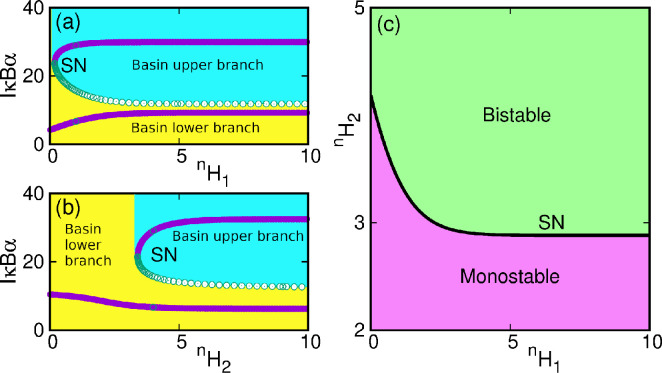
(a) Single-parameter bifurcation diagram with ${,}^{n}H_{1}$ for ${,}^{n}H_{2}=4$. (b) Single-parameter bifurcation diagram with ${,}^{n}H_{2}$ for ${,}^{n}H_{1}=1$ both showing a saddle-node bifurcation, which causes the bistability. (c) Two-parameter bifurcation diagram with ${,}^{n}H_{1}{-}^{n}H_{2}$. The black line is the saddle-node bifurcation line separating the mono- and bistable regions.

We further performed two-parameter bifurcation analyses for our model system as depicted in figure 2c,d. Here, the monostable states (shown in green shade) are separated from the bistable ones (purple shade) by the SN lines. The main findings from this analysis are that we can figure out the region corresponding to the monostable and bistable, a line separating them, and an identification of the *cusp point*, if any. The *cusp point* in a two-dimensional bifurcation diagram shows a signature of catastrophic change in the system. It is a bifurcation of equilibria in a two-parameter family of dynamical equations at which the critical equilibrium has one zero eigenvalue, and the quadratic coefficient for the SN bifurcation vanishes. At the cusp bifurcation point, two branches of SN bifurcation curves meet tangentially, forming a semicubic parabola. We find from our two-dimensional bifurcation diagram in panel (c), which delineates a bistable region in the $k_{d}-$NF-κB parameter space. Here, we observed a *cusp point* at the origin. We find another *cusp point* (CP) in the $k_{1}$ and $k_{d}$ parameter space in figure 2d, a signature of catastrophic change we could observe in the system. We also investigate the system’s dynamics concerning the Hill coefficients ${,}^{n}H_{1}$ and ${,}^{n}H_{2}$, respectively. The bifurcation diagram with ${,}^{n}H_{1}$ is shown in figure 3a. The same for varying ${,}^{n}H_{2}$ is shown in figure 3b. From the figures, it may be noted that the bistability arises due to an SN bifurcation in the system. The stable fixed points (solid points) in the upper and lower branches are separated by unstable points (hollow points). We vary both ${,}^{n}H_{1}$ and ${,}^{n}H_{2}$ and plot in a two-parameter bifurcation diagram in ${,}^{n}H_{1}{-}^{n}H_{2}$ space in figure 3c. Here, the solid black line indicates the SN bifurcation curve. Thus, these parameters are sensitive and play a crucial role in the system’s dynamics.

As expected, the bistability causes bimodality in gene expression. Moreover, low and high expression levels decide the phenotypes of an identical cell. In this regard, a common consensus among geneticists and cancer biologists suggests that multimodality is a crucial aspect of gene expression, and every peak (mode) is linked with the specific physiological state. Distribution with two or more distinguishable peaks implies that transcriptional regulation of genes of interest is conditional (disease-related), a survival mode for some cancers [76]. Numerous studies reveal that there is a direct connection between bimodal gene expression and breast cancer, and it is a matter of ongoing research to list a few enriched breast cancer-associated genes such as ESR1, ERBB2, etc. [16,17,19,29,57–59]. They participate in many pathways and processes of carcinogenesis, which one can employ as markers in breast cancer progression [77]. Various other phenomena encompassing chromosomal rearrangement, gene mutations, multimodal gene expression and post-translational modifications have been identified to alter NF-κB activity, which can be linked with the bimodal expression of IκBα [3,64]. In a nutshell, bifurcation analysis provides information about the existence of a unimodal and multimodal distribution in the parameter space of interest. Also, the study of the typical delay in the transcription process or time-delayed gene activation due to the occupancy of NF-κB on DNA-binding sites offers an insightful view of intricate regulatory pathways behind the modulated gene expression.

#### 3.1.2. Time-delayed dynamics: occurrence of oscillations

**Figure 4.**
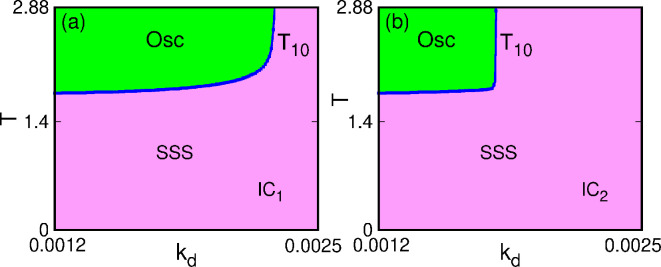
Plotting of $T_{mj}$ for $T_{10}$ obtained analytically shown in the in $k_{d}-T$ space. The blue line with dots indicates the Hopf points in the $k_{d}-T$ stability space along the (a) lower branch and (b) upper branch of figure 2a.The blue points indicate the Hopf points. The purple (darker) zone indicates the stable steady state (SSS) and the green (lighter) indicates the oscillatory (Osc) zone.

We analysed the stability of the finite time-delayed system analytically. The oscillation emerges in the system through the Hopf bifurcation. A detailed analysis of the existence of the Hopf bifurcation is presented in the electronic supplementary material. We calculate the delay for our system (electronic supplementary material, equation S24, section IIB). We plot the $T_{mj}$ from the electronic supplementary material, equation S24, in figure 4 for $T_{10}$. We identified the stable steady-state region for our system in the parameter space (electronic supplementary material, theorem II.2(4)), which is shown by the purple (darker) region in figure 4, and the oscillatory behaviour of the system is denoted by the green region. As the system shows bistability for a range of $k_{d}$ values, we target both the lower and upper branches (figure 2a) in the analytical analysis. Figure 4a shows the $k_{d}-T$ parameter space for the upper branch, and that in figure 4b shows for the lower branch, respectively. It may be noted that in the upper branch, the extent of the oscillatory region is greater than in the lower branch. Physically this means that if the initial concentration of IκBα (NF-κB) is lower, then the extent of $k_{d}$ is lower for emergence of oscillations in the system and vice versa.

For the visual representation of the theoretical results and the effects of time delay on the system equation (2.4), we carry out the numerical simulations. The inclusion of time delay causes the system to experience a Hopf bifurcation, and eventually, oscillations emerge in the system in the form of a limit cycle. We use the same parameter set as the non-delayed case. Inserting these parameters in equation (2.4) and considering ${G^{*}}^{*}={G^{*}}^{*}(t-T)$, we find the positive equilibrium points as $({G^{*}}^{*},I^{*})=$(0.90796,29.50876). The minimum delay time for the occurrence of the Hopf bifurcation is obtained from electronic supplementary material, equation S24, and it is $T_{H}=\text{min}(T_{mj})=1.8$.

**Figure 5.**
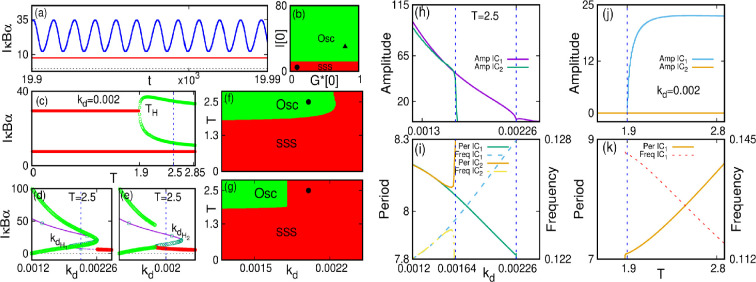
Time series and bifurcation scenarios of system equation (2.4). (a) Time series of oscillation due to delay effect for $k_{d}=0.0015{\text{min}}^{-1}$ and T=2.5 and IC${,}_{1}$ and the stable steady state (red line) for IC${,}_{2}$ (this is drawn along the broken vertical lines in (c–e) and black dot in (f,g)). (b) Basin of attraction in $G^{*}(0)-I(0)$ space, (c) single-parameter bifurcation diagram with time delay T for $k_{d}=0.0015$. The Hopf bifurcation occurs at $T_{H}=1.8$ and oscillation emerges for IC${,}_{1}$; for IC${,}_{2}$ there is no Hopf bifurcation and stable steady state of the lower branch is shown by red line, (d) single-parameter bifurcation diagram with $k_{d}$ for T=2.5. The inverse Hopf bifurcation occurs at $K_{d_{H}}=0.00226$ for IC${,}_{1}$, (e) single-parameter bifurcation with $k_{d}$ and T=2.5 for IC${,}_{2}$, (f,g) Two-parameter bifurcation diagram in $k_{d}-T$ space for IC${,}_{1}$ and IC${,}_{2}$, respectively. (h–k) Variation of amplitude, period and frequency of IκBα with $k_{d}$ for T=2.5, and (j,k) same with T for $k_{d}=0.0015$. For other parameters and details, see text.

Equation (2.4) is further numerically integrated using the fourth-order Runge–Kutta algorithm with a step size h=0.001. We are interested in finding the system’s stable oscillation, so we discard many initial iterations during integration as transients. We use the initial conditions IC${\;}_{1}\equiv \left[G^{*}(-T\to 0),I(-T\to 0)\right]=$$\left[G^{*}(0),I(0)\right]=$[0.8,30.0] (targeting the upper branch) and IC${\;}_{2}\equiv \left[G^{*}(-T\to 0),I(-T\to 0)\right]=$$\left[G^{*}(0),I(0)\right]=$[0.1,5.0] (targeting the lower branch) for this. In figure 5a, we show the time series of IκBα. The figure clearly shows that the number of IκBα protein oscillates in the limit cycle with time. The limit cycle oscillation (arising out of the IC${,}_{1}$) is shown for $k_{d}=0.002$ and T=2.5, i.e. for the broken vertical lines in figure 5c,d and the black dot in figure 5f. For IC${,}_{2}$, the system does not exhibit oscillations and rests in the stable steady state shown by the red line in figure 5a.

It is interesting to note that the system does not oscillate for all initial conditions. Instead, we find oscillatory behaviour for a certain range of initial conditions. Otherwise, the system stays in a stable steady state. To identify the initial conditions, we show the basin of attraction for $k_{d}=0.002$ and T=2.5 as shown in figure 5b. For lower values of I(0) (denoted by red zone), the system stays in the stable steady state, and for larger values (green zone), the system becomes oscillatory. The result demonstrates that the system must possess a minimum initial threshold concentration value of IκBα below which we do not find any oscillation. Moreover, it is worth noting that the initial concentration of NF-κB does not affect the oscillation. The IC${,}_{1}$ and IC${,}_{2}$ are denoted by the solid triangular and circular points, respectively, in this figure.

We further study the bifurcation scenarios in the presence of time delay. For the present system, the bifurcation diagrams suggest that in the presence of time delay, the system shows much richer dynamics, including limit cycle oscillations and stable steady state, which cannot be anticipated in a non-delayed system. We investigate the bifurcation scenarios in single- and two-parameter spaces as described below.

In figure 5c–g, we show the relevant bifurcation scenarios. Figure 5c shows the single-parameter bifurcation diagram with time-delay T as the control parameter for $k_{d}=0.002$ and IC${,}_{1}$. Here, it may be noted that for lower values of T, the system stays in the upper stable steady state, indicated by the red line in the figure. The state is equivalent to the upper branch of states in figure 2a,b. An increase in T causes the system to experience a Hopf bifurcation at $T_{H}=1.8$, and a stable limit cycle emerges. The stable limit cycle is shown by the green line in figure 5c. The bifurcation diagram is drawn by identifying the local extrema of the time series. For IC${,}_{2}$, the variation of time delay does not cause Hopf bifurcation and the system rests in the lower stable steady state as shown by the red line in figure 5a,c.

The effect of variation of the control parameter $k_{d}$ is analysed and shown in figure 5d,e, respectively, for IC${,}_{1}$ and IC${,}_{2}$ for T=2.5. For IC${,}_{2}$, the system emerges oscillations and at $k_{d_{H_{2}}}=0.00226$, it experiences an inverse Hopf bifurcation along with an SN one, which causes the system to bring to the lower branch stable steady state. For IC${,}_{2}$, the system shows oscillations up to $k_{d_{H_{1}}}=0.00182$. At $k_{d_{H_{1}}}$, it experiences an inverse Hopf and SN bifurcation and goes over to lower branch stable steady state (shown by the red line in figure 5e). In figure 5d,e, we also plot the bifurcation for non-delayed case (the blue dots), and it confirms that the upper branch of the stable steady state loses its stability through Hopf bifurcations and oscillations emerge.

Finally, we summarize the system dynamics in the two-parameter ($k_{d}-T$) bifurcation space in figure 5f,g. Here, the red zone indicates the stable steady state, and the green zone indicates the oscillatory zone. Overall, our analysis shows that Hopf bifurcation separates these zones. Figure 5f shows the scenario for IC${,}_{1}$, i.e. along the upper branch of fixed points in figure 2a. Figure 5g shows the same for IC${,}_{2}$ (along the lower branch in figure 2a). It may be noted that the results obtained in numerical simulation (figure 5f,g) and those obtained in the analytical method (figure 4a,b) match well. Here also the oscillatory region for the lower branch is narrower than that for the upper branch.

The effect of the system parameter ($k_{d}$) and time delay (T) on the oscillation amplitude, period and frequency of the system is studied and shown in figure 5h–k. The quantities corresponding to the upper branch of stable steady state in figure 2a are represented by ‘Amp IC${,}_{1}$’, ‘Per IC${,}_{1}$’ and ‘Freq IC${,}_{1}$’ and those along the lower branch are represented by ‘Amp IC${,}_{2}$’, ‘Per IC${,}_{2}$’ and ‘Freq IC${,}_{2}$’ in figure 5h–k, respectively. With an increase in $k_{d}$ (for T=2.5), the amplitude and period of IκBα decrease, and frequency increases, as shown in figure 5h,i. Thus, increasing $k_{d}$ causes the system to oscillate at larger frequencies and decreased amplitude and period. On the other hand, with increasing T (for $k_{d}=0.002$), the amplitude of IκBα increases and becomes fixed for higher values of T for IC${,}_{1}$, and the period increases (frequency decreases). For IC${,}_{2}$, there are no oscillations and the system amplitude is zero. The scenario is shown in figure 5j,k. Thus, increasing T causes the system to oscillate with fixed amplitude and reduced frequencies.

**Figure 6.**
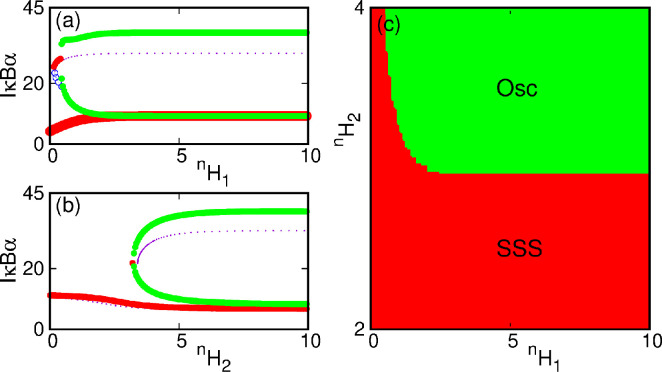
(a) Single-parameter bifurcation diagram with ${,}^{n}H_{1}$ for ${,}^{n}H_{2}=4$, (b) single-parameter bifurcation diagram with ${,}^{n}H_{2}$ for ${,}^{n}H_{1}=1$ both showing there is a saddle-node and a Hopf bifurcation at the upper branch. The green line indicates oscillations and the red line stable steady state. The dots with purple colour are the upper branch for non-delayed system (figure 3, which becomes unstable in the presence of time delay T=2.5). (c) Two-parameter bifurcation diagram with ${,}^{n}H_{1}{-}^{n}H_{2}$ distinguishing stable steady state (red) and oscillatory (green) zones.

In the presence of time delay, we investigate the system dynamics with the Hill coefficients ${,}^{n}H_{1}$ and ${,}^{n}H_{2}$. We draw the bifurcation diagram with ${,}^{n}H_{1}$ in figure 6a. Here, it may be seen that the upper branch (drawn by the purple dots) becomes unstable, and oscillation (green points) emerges through a Hopf bifurcation. A similar bifurcation with ${,}^{n}H_{2}$ is shown in figure 6b. Here, the upper branch becomes unstable, and oscillation (green points) emerges through a Hopf bifurcation. The two-parameter bifurcation in the ${,}^{n}H_{1}{-}^{n}H_{2}$ space is shown in figure 6c. Here, the red zone indicates the stable steady state, and the green zone indicates the oscillatory regions. Hence, from figures 3 and 6, it may be concluded that in the presence of time delay, the system is bistable, but rather than two fixed points, there exists one fixed point and one oscillation.

The time delay associated with gene activation offers an intriguing mechanistic pathway for the modulation of gene expression. However, for an insightful view, we consider stochasticity associated with the GRN of interest in our model. A fundamental question in biology is how normal cells in various organisms perform their task efficiently despite encountering noisy gene expression, which produces *phenotype diversification*. Cells confront intrinsic noise and environmental noise that fluctuate over time. Intrinsic noise is innate to the system under consideration and is associated with stochastic molecular interactions, e.g. inherent uncertainty about the reaction time associated with the transcriptional processes.

### 3.2. Results obtained from stochastic dynamics

#### 3.2.1. Effect of coloured noise

**Figure 7.**
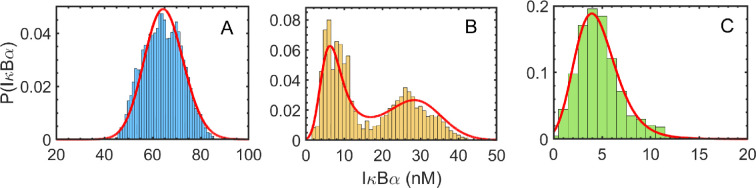
Comparison between the probability densities obtained from the solution of the reduced Fokker–Planck equation (2.6) (solid line) and Gillespie's simulation (histogram). Panels A and C are for the monostable regions, and B indicates the bistable region, which can be well correlated with the bifurcation diagram. The values of the degradation parameter $k_{d}$ for panels A, B and C are $10^{-3}{min}^{-1}$, $2\times 10^{-3}{min}^{-1}$ and $3\times 10^{-3}{min}^{-1}$, respectively.

Bifurcation analysis in figure 2 points out the ability of a system to exhibit bistability; thus, exploring its dynamics in the presence of noise will be interesting. We perform stochastic dynamics of this system in the presence of white and colour noises. Specifically, we calculate the SSPD results using our theoretical analysis. Figure 7 is the SSPD for non-fluctuating protein degradation rate parameters obtained from equation (2.6) and KMC simulations. We consider three different values of $k_{d}$, namely, 10${,}^{-3}$, $2\times 10^{-3}$ and $3\times 10^{-3}$ chosen from bifurcation analysis in figure 2. We observe a fair correlation between the results obtained from the reduced master equation and the KMC simulation. We find bimodality in the protein’s production over a range of protein degradation parameters, $k_{d}$. We find similar results by varying the concentration of NF-κB. The results correlate well with the bifurcation analysis, showing that the degradation rate parameter can induce bimodality that may be related to the *cellular decision-making* for this regulatory network. It thus becomes a significant step to identify the parameters space, which can control the phenotypic states and thus demands precise attention towards a better understanding of complex degradation mechanisms associated with such subtle gene regulatory architectures.

In most analyses, the SSA does not consider the time-varying rate parameter between two successive reactions. However, this is only sometimes true for reactions undergoing multistep pathways, and several studies have been reported analysing stochastic models with fluctuating rate parameters [29,30,45,71]. Thus, we considered the role of coloured noise on the degradation rate $k_{d}$. A close comparison between the SSPD obtained from UCNA analysis in the regime of colour and white noises for the variation of *noise strength,* i.e. *D* is presented in the electronic supplementary material, figure S1. We observed that the strong noise (D=3) leads to the broadening of the SSPD function, which signifies its importance in biological systems. Broadening in the distribution function may be related to the multiple phenotypes observed in biological systems. Usually, biological systems are linked with noises of varying strengths that can fine-tune cellular outcomes/decisions by randomly turning on gene silencing/activation or burst expressions. The randomness associated with biological reaction events gives rise to biological noise, which may be one of the origins of *cell variability* [15,29,78]. Thus, exploring the noise-induced phenotypes at the single-cell level is essential for the correct understanding of such cell variability.

**Figure 8.**
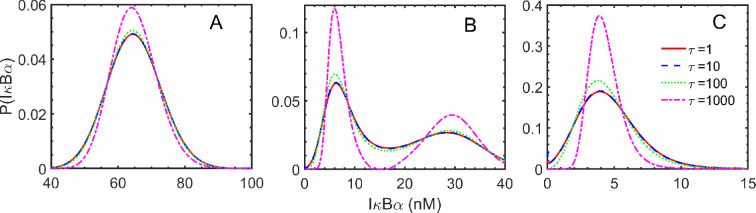
Probability versus IκBα plots obtained from UCNA analysis in the regime of colour and white noises for the variation of *correlation time,τ* . Panels A and C are for the monostable regions, and B indicates the bistable region, which can be well correlated with the bifurcation diagram. The values of the degradation parameter $k_{d}$ for panels A, B and C are $10^{-3}{min}^{-1}$, $2\times 10^{-3}{min}^{-1}$, and $3\times 10^{-3}{min}^{-1}$, respectively.

We then investigated and presented the impact of *correlation time,* i.e. on SSPD obtained from UCNA analysis in the regime of colour and white noises in figure 8. We find that a strong correlation (τ=1000) increases the sharpness in the distribution function, which may be lined up with the multiple phenotype diversity as commonly observed in biological systems. In general, biological systems are associated with noise and are responsible for different phenotype behaviours, e.g. noise can affect the cell fates by randomly turning on either latency or reactivation [32]. Thus, correlation time assists in finding genetic behaviour at the single-cell level to fine-tune cell phenotype robustness. Our analysis shows that tuning the D and τ, i.e. pinpointing where and how much the biological system of interest needs to be perturbed to control a desired phenotype.

**Figure 9.**
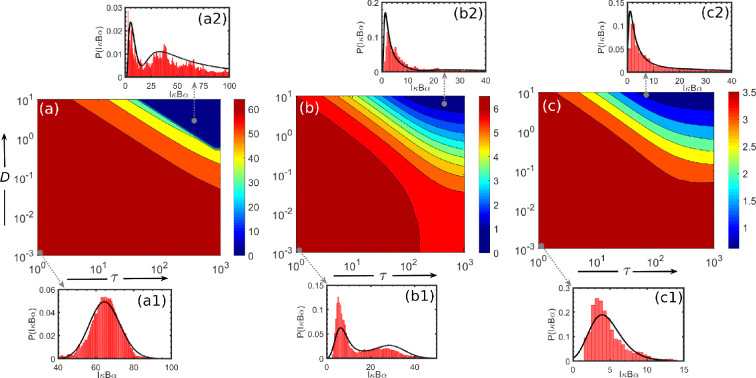
Two-dimesnional contour plot presenting the maximum probability value or mode of the IκBα protein distribution ($P_{max}$ (IκBα)) obtained from UCNA analysis in the regime of colour and white noises in the D−τ space. The values of the degradation parameter $k_{d}$ for panels (a), (b) and (c) are $10^{-3}$, $2\times 10^{-3}$ and $3\times 10^{-3}$, respectively. Panels (a1), (a2), (b1), (b2), (c1) and (c2) represent SSPD results for the production of IκBα protein wherein black-coloured solid lines represent results obtained from UCNA, and red-coloured histogram indicates results obtained from the stochastic simulation algorithm.

Furthermore, we present the two-dimensional contour plots in figure 9 for the mode of the produced protein, IκBα protein as obtained from UCNA analysis in the D−τ space. The value of the degradation parameter $k_{d}$ for panels (a), (b) and (c) are $10^{-3}$, $2\times 10^{-3}$ and $3\times 10^{-3}$, respectively. Marked insets (a1), (a2), (b1), (b2), (c1) and (c2) are a few representative probability distributions for the production of IκBα protein at a particular value of D and τ. The black-coloured solid lines represent results obtained from UCNA, and the red-coloured histogram indicates results obtained from SSA. It can be seen from the results that a fair correlation exists between results obtained from theoretical and numerical approaches. From panels (a), (b) and (c), it becomes clear that for high values of D and τ, the $P_{max}$ (IκBα) value decreases or shifts in the position of the peak we are observing. As the extrinsic fluctuations (environmental noise) are associated with the degradation rate parameter, which decides the lifetime of fluctuations in IκBα protein number; therefore, the extrinsic timescale merges with the intrinsic timescale, thereby resulting in the shifting of the mode of the IκBα protein distribution [79]. Interestingly, panel (a) shows that other than $k_{d}$ and N parameters, the values of D and τ are also other critical players for inducing bimodality into the system, which is commonly known as *noise-induced bimodality* (NIB) [33,45,78]. This property of exhibiting bimodality as the D and τ become high is correct for unimodal parameter sets in the vicinity of bimodal parameter sets in the parameter space. The shift of the system from an unimodal to bimodal distribution is caused by the random stochastic fluctuations, which are high enough in the degradation rate parameter. In a nutshell, this endemic nature of the environmental noise experienced by the cell in biological systems is responsible for producing *gene expression variability* in the cellular state space, which could lead to phenotypic diversification across individual cells or populations of cells.

The obtained SSPD from the UCNA method is presented in figure 9 for a range of *noise strength* (D) and *correlation time* τ. We observed that the SSPD becomes narrow and broadened as the respective correlation time, τ (figure 8), and noise strength, D (electronic supplementary material, figure S2) increases. Moreover, we observed from panel (a) (figure 9) that other than $k_{d}$ and N parameters, the values of D and τ are also significant parameters for inducing bimodality in the population of IκBα at large D and τ values. Thus, it is crucial to determine the role of coloured noise near the boundary between monostable and bistable regions. We chose the protein degradation parameter value, $k_{d}=0.0024$, which considers the boundary between the monostable and bistable region, as shown in figure 2c.

In the electronic supplementary material, figure S2, we vary the value of D and τ with the parameter sets in panels (a) and (d) in the monostable region, which is in the proximity of the bistable region and is marked with red-coloured solid lines for the lowest values of both D and τ. We observed from panel (a) that for low values of correlation time (τ), no matter how high the noise strength (D) is, the monostable SSPD does not change. On the contrary, in panel (d) for low values of D, if we consider the large value of τ, we could see the feature of NIB. Furthermore, for high values of D and τ, we observed that SSPD changes from a monomodal to a bimodal and shifts the peak positions, as shown in panels (b) and (c). The reason behind NIB can be correlated with the large fluctuations in the degradation rate parameter, which leads to occasional jumps between the unimodal and bimodal regions. Understanding this unique noise feature that induces bimodality through an intricate degradation mechanism is essential from a *cellular decision-making* perspective [33,45]. Thus, noise offers another regulatory pathway for cells to influence the *phenotypic states*, which could be of great importance for synthetic biologists to influence cellular outcomes through complex degradation mechanisms. Also, the shift in the position of the peak corresponding to maximum IκBα expression provides an ample opportunity to regulate various physiological responses, e.g. telerogenic trait of antigen cells when exposed to various evolving growth factors [80].

#### 3.2.2. Power spectra analysis

**Figure 10.**
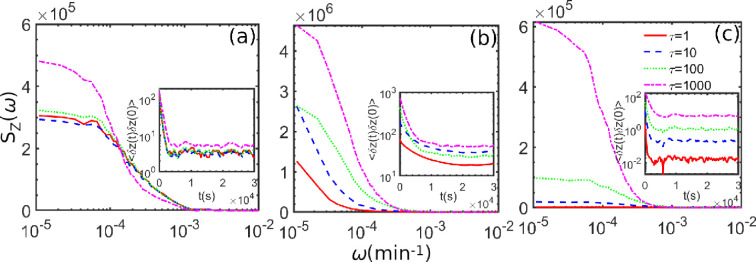
Here, we present the power spectral density for the different *correlation time*, i.e. τ values. We report the results after the incorporation of coloured noise using UCNA in the three different regimes of the bifurcation curve in panels (a), (b) and (c), where $k_{d}$ values are $1\times 10^{-3}$, $2\times 10^{-3}$ and $3\times 10^{-3}$, respectively. Here, the solid red line represents the power spectral analysis in the non-coloured degradation rate parameter regime. We have presented the power spectral density and auto-correlation (in the insets) results after taking the average of 1000 stochastic trajectories to produce IκBα protein.

A robust relation between the power spectra of IκBα protein number fluctuation and the underlying biomolecular mechanism could be employed to investigate the dynamics of IκBα protein fluctuations. Thus, we calculate the power spectra to assess the effect of coloured noise on the stochastic dynamics of protein production. The frequency spectrum, $S_{z}(\omega )$, is equivalent to the Fourier transform (FT) for the steady-state time correlation function of the product number fluctuations and is given by


(3.1) $$\begin{array}{lcr} & S_{z}(\omega )=\int_{-\infty }^{\infty }dte^{-2\pi i\omega t}\langle \delta z(t)\delta z(0)\rangle_{ss}, & \end{array}$$

where the symbol z is a random variable corresponding with the number of produced protein IκBα, δz(t)=(z(t)−⟨z⟩) is the deviation of the protein number from its mean value at time t. The symbol ⟨⋅⟩ is the average over many trajectories. The functional form of the $S_{z}(\omega )$ or the correlation function of the produced IκBα depends on the mechanisms involved during its production. The auto-correlation function and the power spectrum characterize the role of coloured noise in fine-tuning the IκBα protein’s production levels for stochastic gene expressions. We examine how these short-range correlated noises influence the power spectra of protein production. The power spectrum and correlation function are analysed for the different values of $k_{d}$, τ and D as shown in figure 10 and electronic supplementary material, figure S3. We notice that the correlation functions decrease monotonically, indicating that the correlation diminishes monotonically with time. We find relatively slow decay in correlation in the bistable region (in panel (b)) compared to the two monostable regions (in panels (a) and (c)). The power spectra obtained from the correlation function’s FT provide a low-frequency band for all cases. The low-frequency band in the power spectrum of protein production during gene regulation refers to the frequencies below a certain threshold. The specific system under study determines this threshold and can vary depending on the cellular conditions.

In figure 10 and electronic supplementary material, figure 3, we vary the parameters τ and D to examine the auto-correlation and power spectral density. We presented correlation results in the insets of figures and observed exponential decay in correlation. However, we notice fast decay when choosing a short protein lifetime, i.e. a high $k_{d}$ value (in panel (c)). We observed in the inset of figure 10 that for a high value of *correlation time,* i.e. τ, we observed slow decay, which suggests the role of memory (i.e. non-Markovian effects), in impacting correlation and power-spectra results. These analyses also support our observation in figure 8, which indicates an increase in narrower protein production region with an increase in the τ value. We furthermore varied D in the electronic supplementary material, figure S3 and observed that for a high *noise strength,* i.e. D value, the decay is fast, which again supports our analysis in the electronic supplementary material, figure S2, indicating broader protein production region with an increase in D value. Thus, our analyses align with the various theoretical and experimental observations that suggest the role of memory in impacting cellular fate decisions [15,81].

### 3.3. Stochastic analysis of switch-like gene expression driven by NF-κB-mediated SEs and TEs

Our proposed model considers the switching of the promoter states between active ($G^{*}$) and inactive (G) through the binding of NF-κB (N) and IκBα (I) protein. The model considers oligomerization through Hill’s coefficients ($n_{H_{i}}$) (i=1,2) present in Hill’s function, $f_{A}$ and $f_{R}$. The strength of binding or the binding affinity between the NF-κB and promoter region of DNA is characterized by the parameter, $K_{m_{1}}$. Note that an increase in N results in binding a cluster of proteins at the promoter region. The binding of clusters of proteins to the gene’s promoter region enhances the expression abruptly, which is quite distinct from the regular binding of TF to the promoter region. The binding of such a cluster of proteins to the promoter region is defined as an SE, and the regular protein-promoter interaction is called a TE. Gene expression via SE produces higher fold change than the expression controlled by the TEs [4,61].

**Figure 11.**
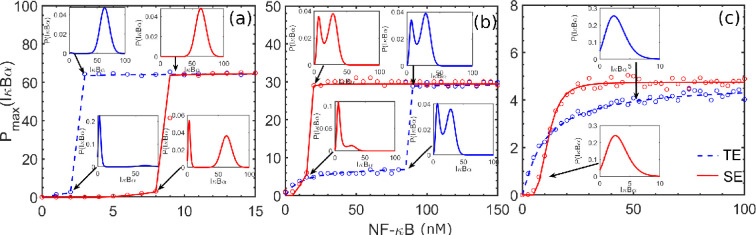
IκBα value corresponding to maximum probability or mode of the IκBα protein distribution ($P_{max}$ (IκBα)) produced via SE- and TE-mediated gene expression: The dashed blue and solid red colour lines indicate the TE- and SE-mediated gene expression, respectively. Here, circles indicate the simulation results obtained from stochastic analysis. The values of the degradation parameter $k_{d}$ for panels a, b and C are $10^{-3}$, $2\times 10^{-3}$ and $3\times 10^{-3}$, respectively.

Literature suggests [61] that the typical values of $n_{H_{i}}$ for the expressions mediated by TE and SE have values ≈ 1 and 4, respectively. Therefore, we use these numbers to model them as a function of NF-κB concentration. The results are presented in figure 11, where we plot the IκBα value corresponding to the maximum probability value or mode of the produced protein, $P_{max}$ (IκBα). These results show the direct impact on the phenotypic switching mediated via NF-κB. Literature suggests that phenotypes of the identical cell population are influenced by the states corresponding to low and high expression levels, and calculation of the mode of the protein’s distribution function can infer this detail efficiently [79,82].

We observe an abrupt switching in the expression level for the TE (blue colour) and SE (red colour) in panels (a) and (b) for the variation of concentration of NF-κB, whereas no such effect was observed for panel (c). These results show connectivity with the bifurcation analysis because figure 2c shows that for higher values of $k_{d}$, there exists only monostable region, no matter how high the NF-κB concentration. Furthermore, we show the distributions of produced protein in the subplots of figure 11. It clearly shows the bimodal distributions of the protein in panels A and B, and a unimodal distribution of protein was observed in panel C. It is also clear from our analysis that the results obtained from UCNA-based theory and the results obtained from SSA are fairly correlated with each other.

Thus, it is pointed out that when NF-κB binds as a cluster to promoter regions, a significant difference in gene expression level is exhibited for a specific range of NF-κB concentrations. Our observed results correlate well with the earlier results of Michida *et al*. [61], who reported that interactions among enhancer region and NF-κB molecules could be cooperative as well as non-cooperative. These types are chosen based on the amount of NF-κB present in the system and chromatin accessibility [61]. Based on a similar argument, figure 11 supports the role of NF-κB as SEs and TEs, where SE produces a high expression level relative to TE.

Our analysis thus reveals that the concentration of NF-κB binding to SE describes the features of switch-like expression or phenotypic switching and also impacts the gene expression profiles robustly [61,83]. Thus, our analysis indicates that the transcriptional regulation of downstream genes mediated via NF-κB could involve liquid condensate formation through macromolecular interactions. This distinct expression mediated via TE and SE leads to *cellular variability* in the transcriptional response. We also observed the existence of bistability in this system. Bistable or multistable features in a GRN are essential for *cellular decision-making* and often correlate well between genotype and phenotype relations. Thus, we infer from this analysis that the genes can tune their expression by forming clusters of transcription-regulating proteins at the enhancer elements of a gene.

## 4. Conclusion

In summary, we have studied non-delay and delay dynamics for synthesizing protein IκBα using our proposed model. We also consider environmental variability in the dynamics, which we apply on a gene encoding for an IκBα protein and is regulated by NF-κB in this work. We include colour noise into the model as introduced by an OU process at the protein degradation step, as the synthesized protein has a lifetime. The stochastic dynamics of this system become interesting since it exhibits bistability, as revealed by the bifurcation analysis.

Bistability in the deterministic non-delayed system may be correlated with the switching between two functional states or the phenotype diversity of a GRN. The NF-κB system tends to settle into a two stable states marked by the low and high concentration values of both the proteins IκBα and NF-κB. The system also shows bistability, when we chose the $k_{d}$ as a bifurcation parameter. The bifurcation analysis provides information about the existence of an unimodal and multimodal distribution in the parameter space of interest and their transitions. Distribution of two or more distinguishable peaks implies that transcriptional regulation of genes of interest is conditional, which may be linked with diseases such as cancers [76]. Further, the time delay in NF-κB gene activation not only provides a mechanism to induce or eliminate oscillation but also may affect the amplitude, period and hence the frequency of the IκBα oscillations. Experiments suggest that the amplitude and period (frequency) of the NF-κB (and IκBα) oscillations may cause bipolar cell fates that include survival and apoptosis of cancer cells [9,16,57,58,84]. The regulation of NF-κB (and IκBα) through time delay may provide insightful views in cancer treatment and other related diseases. Hence, the time delay in the gene activation may lead to an important potential direction for treating cancer and immunological diseases. The time delay required for the extension and splicing of genes to mature RNA is increased by the transcription and splicing of intron sequences [47,85,86]. Changing the number of introns is an effective way to vary the time delay.

We conducted stochastic dynamics and analysed them using analytical methods and kinetic Monte Carlo simulation of this gene regulatory system. Our analysis considered static and fluctuating rate parameters based on experimental data. Initially, we approximated the system to follow Markovian dynamics and used a static rate parameter for modelling. We solved the corresponding FPE to obtain the SSPD of the produced protein. However, when considering protein lifetime, we included a fluctuating rate parameter driven by coloured noise. It involves the introduction of coloured noise to the rate parameter, including a short-range correlator to address protein degradation at the time (t+τ) if it is produced at the time (*t*). We applied the UCNA method to analyse the system analytically and obtained the SSPD results. For the KMC simulation, we used the time-dependent Anderson’s next reaction method as detailed in the electronic supplementary material, section II.C [87]. Our results from both analytical and numerical methods showed a good correlation. To quantify the effect of coloured noise, we performed power spectral density analysis by Fourier transforming the auto-correlation of the produced protein. Our findings demonstrate that coloured noise significantly influences the dynamics of protein production.

Our analysis revealed that the bimodality of our proposed model arises from various factors, including (i) the concentration of NF-κB, (ii) the degradation rate parameter $k_{d}$, (iii) the gene activation rate parameter $k_{1}$ and (iv) NIB. This bimodal behaviour can be attributed to a negative feedback mechanism similar to the toggle switch [45,77]. Examples of such behaviour can be found in natural systems, so it is important to understand their origins. We identified NIB through our study and made efforts to comprehend the mechanism of such behaviour for NF-κB-mediated gene regulation. Our analyses revealed a strong relationship between the system’s noise and nonlinearities. The input response plays a crucial role in processing signal noise, leading to sufficient signal distortion that converts the unimodal distribution into a bimodal one. Additionally, we observed that the clustering of transcription-regulating proteins influences the switch in the production of IκB protein. Our calculations show that robust dynamical phenotype transitions can arise from external noise, where the genotype–phenotype correspondence evolves in the parameter space.

We finally show that our integrative analysis of TE- and SE-mediated gene expression and environmental variability corroborates the experimental findings. We analysed them using our theoretical and computational results and presented the biological relevance for our system correspondingly. The analytical and simulation approaches mentioned here are not limited to this particular system, but they can also be broadly relevant in biological systems other than NF-κB-specific genes. Thus, our study will help to estimate TE- and SE-mediated expression and the anticipation of transcription responses quantitatively with better accuracy.

## Data Availability

Data and relevant code for these analyses are available at Zenodo [88]. Supplementary material is available online [89].
